# Questioning Seasonality of Neuronal Plasticity in the Adult Avian Brain

**DOI:** 10.1038/s41598-018-29532-1

**Published:** 2018-07-26

**Authors:** Tatyana Pozner, Yulia Vistoropsky, Stan Moaraf, Rachel Heiblum, Anat Barnea

**Affiliations:** 1 Department of Natural and Life Sciences, The Open University of Israel, Ra’anana, 43107 Israel; 2 Department of Stem Cell Biology, Friedrich-Alexander-Universitaet Erlangen-Nuernberg (FAU), Erlangen, 91054 Germany

## Abstract

To date, studies that reported seasonal patterns of adult neurogenesis and neuronal recruitment have correlated them to seasonal behaviors as the cause or as a consequence of neuronal changes. The aim of our study was to test this correlation, and to investigate whether there is a seasonal pattern of new neuronal recruitment that is not correlated to behavior. To do this, we used adult female zebra finches (songbirds that are not seasonal breeders), kept them under constant social, behavioral, and spatial environments, and compared neuronal recruitment in their brains during two seasons, under natural and laboratory conditions. Under natural conditions, no significant differences were found in the pattern of new neuronal recruitment across seasons. However, under artificial indoor conditions that imitated the natural conditions, higher neuronal recruitment occurred in late summer (August) compared to early spring (February). Moreover, our data indicate that “mixing” temperature and day length significantly reduces new neuronal recruitment, demonstrating the importance of the natural combination of temperature and day length. Taken together, our findings show, for the first time, that neuroplasticity changes under natural vs. artificial conditions, and demonstrate the importance of both laboratory and field experiments when looking at complex biological systems.

## Introduction

Seasonal neuronal recruitment has been linked to seasonal behavior. A prominent example is the song control system in the avian brain, where seasonal changes in song behavior are accompanied by changes in the song nuclei in the brain, including seasonal neuronal recruitment, in almost every seasonally breeding songbird species that has been examined^1^. For example, in adult canaries, which are seasonal breeders, neuronal recruitment in the vocal nucleus HVC of males is temporally related to changes in song, with a peak at the end of summer/early fall^2^. It was demonstrated that both the increase of number of new neurons and singing behavior correlate with the regeneration of HVC-RA (robust nucleus of the arcopallium) circuit in breeding birds^3^. The identity of the new neurons had been confirmed by Burd and Nottebohm^4^, and Paton and Nottebohm^5^ showed that these neurons have neurophysiological profiles and clear neuronal anatomy, and that they are incorporated into functional neural circuits.

Brain plasticity is also correlated to migratory behavior in birds. We have found that in passerines, more new neurons are recruited in the migrant reed warbler (*Acrocephalus scirpaceus*) than in the resident Clamorous warbler (*A*. *stentoreus*), in two forebrain regions that are known to process spatial information - the Hippocampus (HC) and the Nidopallium Caudolaterale (NCL), during spring, summer and autumn^6^. In Columbiformes, on the other hand, we found an overall higher neuronal recruitment in these regions in brains of the resident laughing dove (*Streptopelia senegalesis*) than in those of the migratory turtle dove (*S*. *turtur*)^7^. However, regardless of the differences in levels of neuronal recruitment and their directions within each of the tested pairs of species, it is evident that in doves as well as in passerines, there is lower neuronal recruitment in spring compared to other seasons.

A similar pattern of marked seasonality in neuronal recruitment was reported in blackcapped chickadees^8^, where seasonal brain plasticity correlated with the seasonal food storing behavior and the need to acquire spatial information. The lowest recruitment of new neurons in the HC was evident in early spring (February-March) compared to other seasons (August or October). However, later studies in the same species indicated either another seasonal pattern^9^, or no seasonality at all^10^. Sherry and MacDougall-Shackleton^11^, who reviewed these studies, as well as other studies that investigated additional aspects of hippocampal seasonal changes (e.g. volume), provided evidence that these seasonal changes are not under photoperiod control. They suggested that the variation in the seasonal change in the HC of food-storing parids is the result of various experience-dependent effects, such as the intensity of the behavior, stress, or the amount of exercise in captivity. Another comprehensive recent review by Pravosudov *et al*.^12^ on two food-storing species – black capped and mountain chickadees, pointed to inconsistencies between results from different studies regarding the seasonal effect on neuronal recruitment in the HC. However, in both species they found that rates of new neuronal recruitment in the HC were significantly associated with winter climate harshness, with birds from harsher climates having higher neurogenesis rates.

Testing the interactions between behavior and neuronal recruitment is challenging, because it is easy to confound correlation and causation, and difficult to determine the direction of the causal relationships^13^. Do seasonal environmental changes (e.g. day length, temperature) cause changes in behavior, which, in turn, cause changes in brain nuclei that are associated with this behavior? Or do seasonal environmental changes cause changes in brain nuclei, which, in turn, cause changes in behavior? Brenowitz^14^ presents evidence for the latter suggestion, arguing that seasonal changes in the song nuclei in seasonal species are predominantly regulated by hormonal changes, and that the subsequent changes in song behavior play a secondary role in reinforcing neuronal changes. On the other hand, Ball *et al*.^15^ proposed that singing behavior itself could influence the seasonal neuronal plasticity in the song system, through mediation of testosterone. In addition, as already mentioned above, Sherry & MacDougall-Shackleton^11^ suggest that, in relation to the seasonal food-storing behavior, seasonal changes in the HC may be a consequence of the behavior itself.

In any case, whichever seasonal pattern had been found in the brain, in most of the above mentioned studies the explanation was related to a seasonal behavior – migration, food-storing, song production, or reproduction. The assumption was that neurogenesis (i.e. neuronal proliferation, which in birds occurs only in the ventricular zone; VZ) does not vary seasonally, but rather the survival of these new neurons that migrate from the VZ to the HC is the neurogenic process that varies seasonally. This was supported only in the seasonal black-capped chickadees, where neurogenesis was stable throughout the seasons whereas neuronal recruitment varied^16^. However, in many of our previous studies, we used another species - zebra finches (*Taeniopygia guttata*; ZF), which are considered to be non-seasonal, because they breed at any time, and do not exhibit seasonal changes in singing^17^. Their diet is very narrow throughout the year: they eat seeds from a limited number of grass species, and rarely anything else^18,19^. Zebra finches are also very restricted to specific areas all year round, do not travel long distances and do not migrate (reviewed in Zann^17^). Therefore in our outdoors experiments we assumed that if all behavioral, social, and spatial conditions are constant, then neurogenesis, as well as neuronal recruitment, should be stable throughout the year. Accordingly, we stated that “seasonal changes in temperature and photoperiod were unlikely to have affected the outcome of our studies”^20^. Others have avoided that issue altogether by keeping the birds under constant indoor conditions (e.g. Tokarev, *et al*.^21^). However, to the best of our knowledge, this assumption has not been tested yet.

Accordingly, in the present study we tested whether there is a seasonal neuronal recruitment pattern that is not correlated to behavior, and if so – whether this pattern exhibits less plasticity in early spring than in late summer. We used ZF, which, as mentioned above, are non-seasonal breeders that reproduce opportunistically throughout the year whenever conditions are favorable, both in the wild^17^ and in captivity^22^. To avoid effects of singing behavior, we used females, and kept the social, behavioral, and spatial environment constant. Another advantage of females in this respect is that gonadal steroid hormones and corticosterone have no effect on cell proliferation in the VZ^23,24^. We compared neuronal recruitment rates under two natural outdoor seasons and under artificial settings in the laboratory that imitated the natural conditions. In addition, we used unnatural combinations of photoperiod and temperature to study whether the compatibility between these two cues is significant for neuronal recruitment – namely, whether “mixing” of summer temperature with winter day length and vice versa will affect neuronal recruitment compared with exposure to compatible conditions. Lastly, we tested whether any seasonal effect on neuronal recruitment is region-dependent by analyzing three brain regions that process different sensory inputs, as follows: the HC, which is involved in the processing of spatial information^25–27^ and in stress response^28^; the Nidopallium Caudale (NC), which contains auditory relays^29^ and is thought to be involved in vocal communication and integration of auditory information^30–32^, and one of its sub-regions, Nidopallium Caudolaterale (NCL), is considered functionally equivalent to the prefrontal cortex in mammals^33^ and might have a role in working memory and executive function (e.g.^34^); and the Medial Striatum (MSt), formerly known as Lobus parolfactorius (LPO) which is linked to visual perception and associative learning^35–38^. We believe that testing the possibility of a seasonal neuronal recruitment pattern that is not correlated to behavior will add to the open discussion regarding the functional significance of adult neurogenesis and whether it is vestigial or a consequence of active maintenance^14^. This question is also highly relevant for the constantly growing research community working with ZF, in planning of future experiments that are designed to examine mechanisms that regulate neuronal recruitment.

## Materials and Methods

### Experimental design

Female ZF were reared in outdoor breeding colonies at the Meier Segals Garden for Zoological Research at Tel-Aviv University. The birds were banded with numbered and colored plastic rings for individual identification, and at the age of 4–18 months (after reaching adulthood), they were removed from their native colonies and randomly divided into six experimental groups, to achieve the best possible age-matched distribution, according to availability in the different seasons. Each group consisted of 5 females that were housed in a standard cage (65 × 35 × 45 cm).

**Figure 1:**
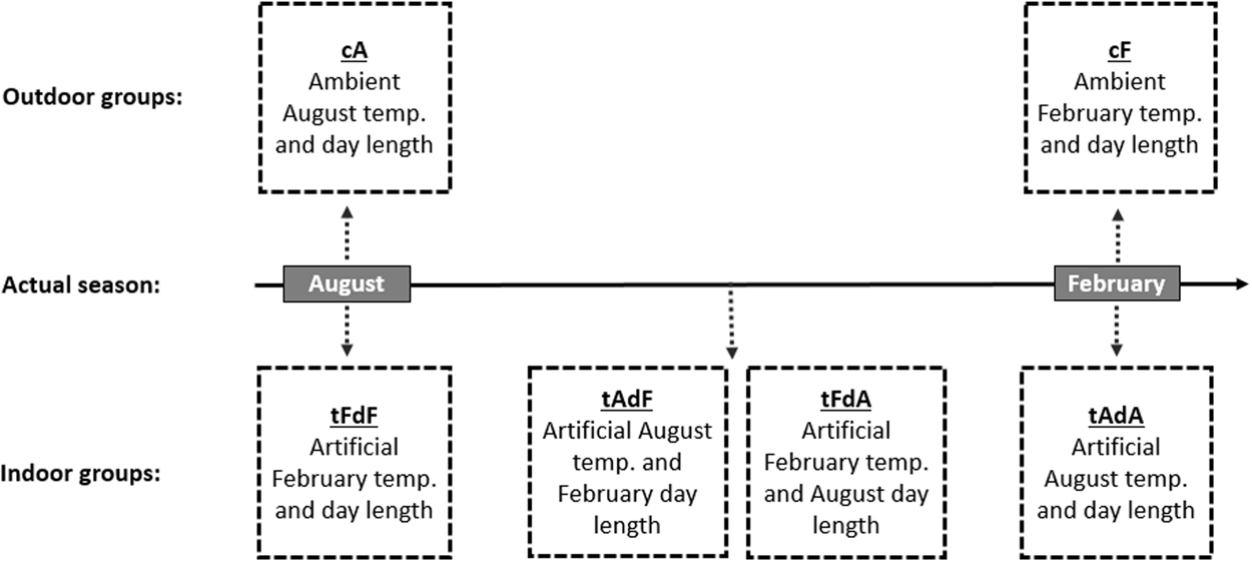
A schematic representation of the six groups that were included in the experimental design, as follows: outdoor ambient August (cA), outdoor ambient February (cF), indoor artificial February temperature and day length (tFdF), indoor artificial August temperature and day length (tAdA), indoor artificial August temperature and February day length (tAdF), and indoor artificial February temperature and August day length  (tFdA). For more details see text.

The six experimental groups are presented in Fig. 1. Two control groups were kept outdoors, one in August and the other in February, and exposed to natural ambient temperature and day length of that season: outdoor control August - cA (mean temperature of 29 °C; mean day length of 13.5hrs), and outdoor control February - cF (mean temperature of 16 °C; mean day length of 11hrs). During each of these months, a group of other females were transferred to indoors cages, where they were exposed to artificial conditions of day length and temperature of the opposing season. Thus, in August the birds that were transferred to indoor cages were exposed to artificial conditions of February temperature and February day length – tFdF (mimicking the conditions in cF), while in February the birds that were transferred to indoor cages were exposed to artificial conditions of August temperature and August day length – tAdA (mimicking the conditions in cA). The “seasonal reversal” in these two indoor groups was done in order to use birds from the same pool, which grew up and kept in identical conditions until the onset of the experiment, and create pairs of reversed groups (i.e. cA vs. tFdF in August; and cF vs. tAdA in February). This design enabled us to simultaneously expose birds, which were randomly chosen from the same pool, to temperature and day length conditions of opposite seasons. Sometime between August and February we transferred two more groups indoors, where they were kept under “mixed” temperature and day length, so that they were exposed to artificial and opposite combinations of temperature and day length: indoor August temperature and February day length – tAdF, and indoor February temperature and August day length – tFdA.

All the six groups were acclimatized to their experimental conditions for 21–24 days, a time period that ensures thermogenic acclimation^39^, and that is also usually accepted for physiological acclimation in birds (e.g.^40–42^). Then, each female received, during two consecutive days, four intramuscular injections of 130 μl (i.e. 100 mg/kg) of bromodeoxyuridine (BrdU; SigmaUltra, diluted 10 mg/ml in sterile water; Sigma). BrdU is a cell birth-date marker, and our dose is the same as previously used to study adult neurogenesis in this species^43–45^. Then, the birds were kept under the same conditions for additional 24 days, a period that allows enough time for neurons born at the time of treatment to migrate to their final destination, undergo final anatomical differentiation^46,47^, and incorporate into functional neural circuits^4^.

### Histology, immunohistochemistry and mapping of brain regions

**Figure 2:**
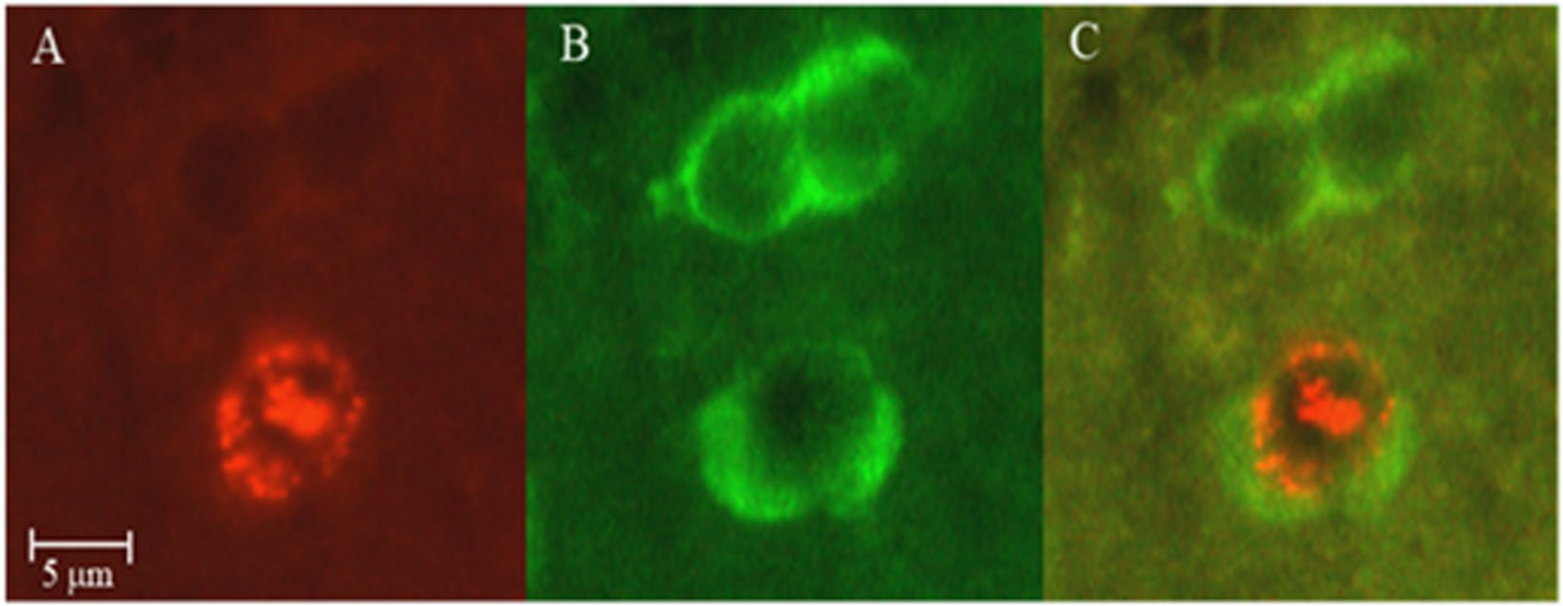
Three microphotographs of the same field, showing a newly formed neuron in the hippocampus of female zebra finch. (**A**) BrdU-labeled cells were identified by their red-stained nuclei; (**B**) All neurons were identified by their green-stained cytoplasm; and (**C**) cells with co-localization of green cytoplasm and a red nucleus were identified as new neurons. (See text for more details). (The picture was taken by S. Barkan and published with his consent).

Twenty four days after BrdU treatment the birds were weighed, killed, their brains were fixed, embedded, cut transversely at 6 µm intervals, and processed for immunohistochemical staining as we described in details previously (e.g.^45^). In short, we used double-labeling fluorescence staining: Hu (a neuronal specific marker) was stained green in the cytoplasm of all neurons, and BrdU (the cell birth-date marker) was stained red in the nuclei of new neurons. Therefore, cells with co-localization of green cytoplasm and a red nucleus were identified as new neurons (Fig. 2).

**Figure 3:**
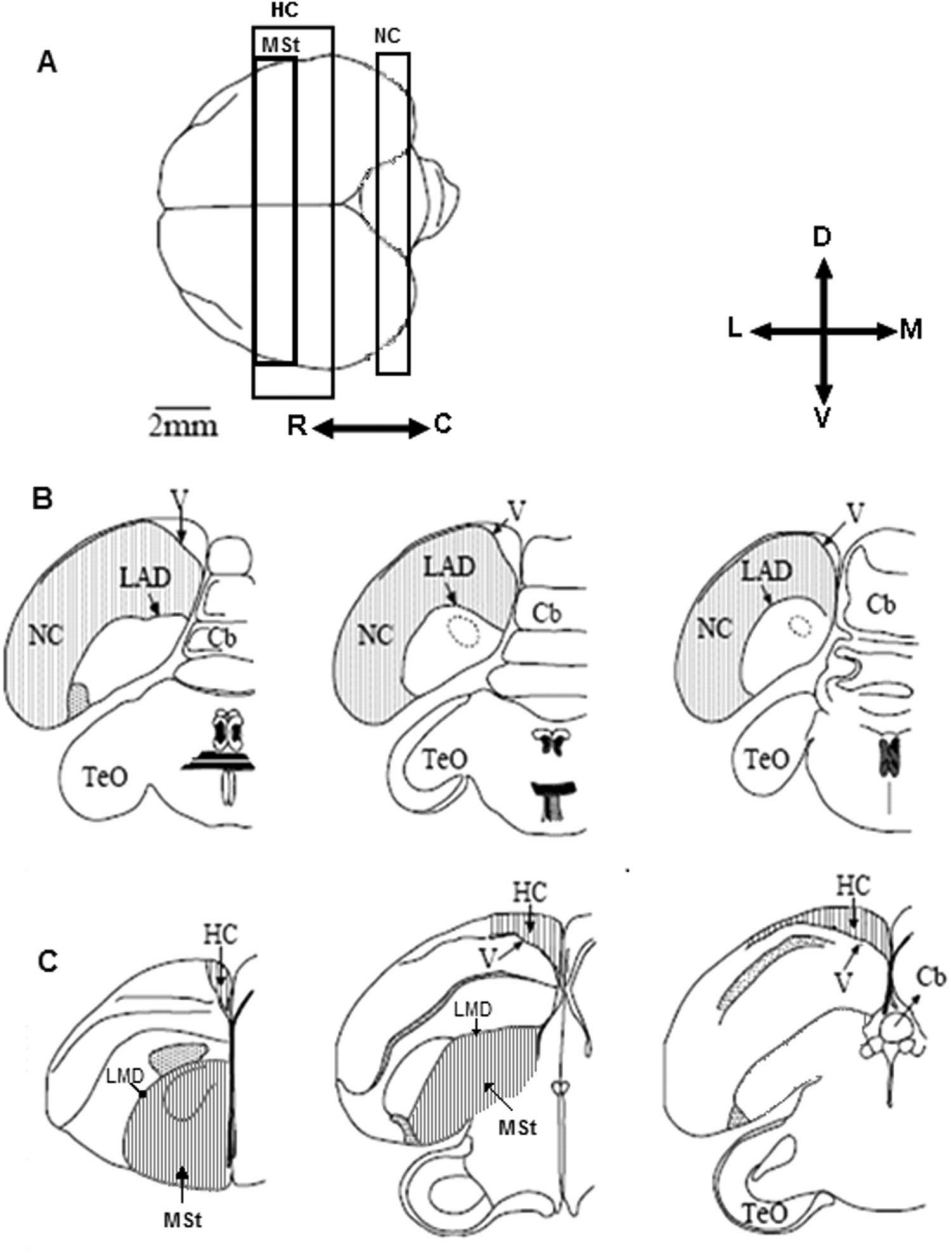
Schematic views of the three investigated brain regions (**A**) Top view of the brain: rostral is to the left, caudal is to the right. We indicate the range within which frontal sections were taken from the Nidopallium caudale (NC), Hippocampus (HC), and Medial Striatum (MSt). Five sections were sampled along the rostro-caudal axis of each brain region. For NC (**B**) and HC (**C**) only three are shown: the most rostral, the middle, and the most caudal (from left to right). For MSt only two are shown: the most rostral and the most caudal (**C**). Abbreviations: Cerebellum (Cb), Lamina arcopallialis dorsalis (LAD), Lateral ventricle (V), Tectum opticum (TeO). Orientations: Dorsal (**D**), Lateral (L), Ventral (V), and Medial (M); adapted from Cattan *et al*.^43^.

As already detailed in the Introduction, we focused our attention on three brain regions (Fig. 3A): The Hippocampal complex (HC), Nidopallium Caudale (NC), and Medial Striatum (MSt). ZF recognize each other and communicate by vocalization and visual information. Hence, because the social and spatial environment were the same and remained constant in all groups, if there is no seasonal effect on neuronal recruitment, one can expect to find similar levels of neuronal recruitment in these regions across groups. However, if neuronal recruitment differs between groups, it will provide an indication of a seasonal effect. Furthermore, the groups in which we created a combination of temperature and day length of different seasons will enable to test possible interactions between these two factors.

All three brain regions have easily recognizable boundaries in transverse sections. To determine the boundaries of NC (Fig. 3B) and those of HC (Fig. 3C), as well as the locations of the five sections that were sampled along the rostral-caudal axis in each of these brain regions, we followed the criteria that are described in Cattan *et al*.^43^. For MSt, the lateral and the dorsal boundaries were defined by lamina medullaris dorsalis (LMD); the ventral boundary was defined by the surface of the brain and the medial boundary was defined by the midline (Fig. 3C). MSt stretches rostro-caudally over a distance of approximately 1200 µm; its most rostral section corresponded to A4.0 in the canary atlas^48^ (Fig. 3C), and its most caudal section corresponded to A1.8 there. Similar to HC and NC, within this distance we sampled five sections along the rostral-caudal axis, with an average interval of 240–360 µm between them.

We used a computerized brain-mapping system (Neurolucida; Stereo Investigator; Micro-BrightField Ltd.) to draw boundaries of the mapped areas, to count labeled and unlabeled neurons, and to quantify other neuronal parameters, as described below. Each of the five sampled sections of HC, MSt and NC were scanned using a 63X objective. In the HC and MSt, which are relatively small regions, we completely scanned all the mapped sections, using the meander scan sampling in our mapping system. In the NC, which is a relatively large brain region, we scanned about 35% of the entire sampled area, by using the fractionator sampling, which chose random sampling squares. From our previous work^43^ we know that this sampled portion yields results that are equivalent to mapping of the entire area. Accordingly, in each mapped section, we recorded the location of all BrdU labeled neurons and counted them. The total number of BrdU labeled neurons per group ranged between 119–603 in MSt, 20–435 in NC, and 15–86 in the HC.

In each brain and for each region, we also measured the nuclear diameters of ten BrdU labeled neurons, and those of a sample of all neurons (labeled and unlabeled), as described in Vistoropsky *et al*.^45^. The values of section thickness and the mean neuronal nuclear diameter of labeled neurons in a certain brain region served to estimate the number of new neurons per mm^3^ in each section and compensate for over-counting [number of cells/mm^3^ = number of cells/area * (T/(T + D)]; where T = section thickness (6 µm, as indicated above) and D = mean nuclear diameter], using the Abercrombie stereological correction equation^49^. Data from all the five mapped sections in each region of each brain were averaged so that each region in that brain was represented by a single estimated number of BrdU labeled neurons per mm^3^.

### Statistical analysis

Numbers of BrdU labeled neurons per mm^3^ were transformed into square root values to achieve a normal distribution^50^. The transformed data were analyzed by repeated measures analyses of variance (ANOVA), in which the fixed factor was the six groups, and brain regions (HC, NC, MSt) served as the repeated measures. This was done because brain regions are considered to be dependent variables - they are all in the same brain of a particular bird, and were found to be significantly (p < 0.0001) correlated^51^. Post hoc Bonferroni test was carried for comparisons of the means. In order to discern the effect of location and seasonality we performed two-way ANOVA on the four groups of natural combinations: cF, cA, tFdF, tAdA. First on all three brain regions by repeated measures and then for each brain region separately. Individual one-way ANOVAs were done for pre-planned comparisons. Alpha was 0.05. JMP® Pro 13 (SAS Institute Inc., Cary, NC) was used for all analyses.

### Ethics Statement

This study was approved by the Tel-Aviv University Institutional Animal Care and Use Committee (permit L-15-013) and was carried out in accordance with its regulations and guidelines regarding the care and use of animals for experimental procedures.

## Results

### An overall comparison between natural, artificial, and “mixed” conditions

**Figure 4:**
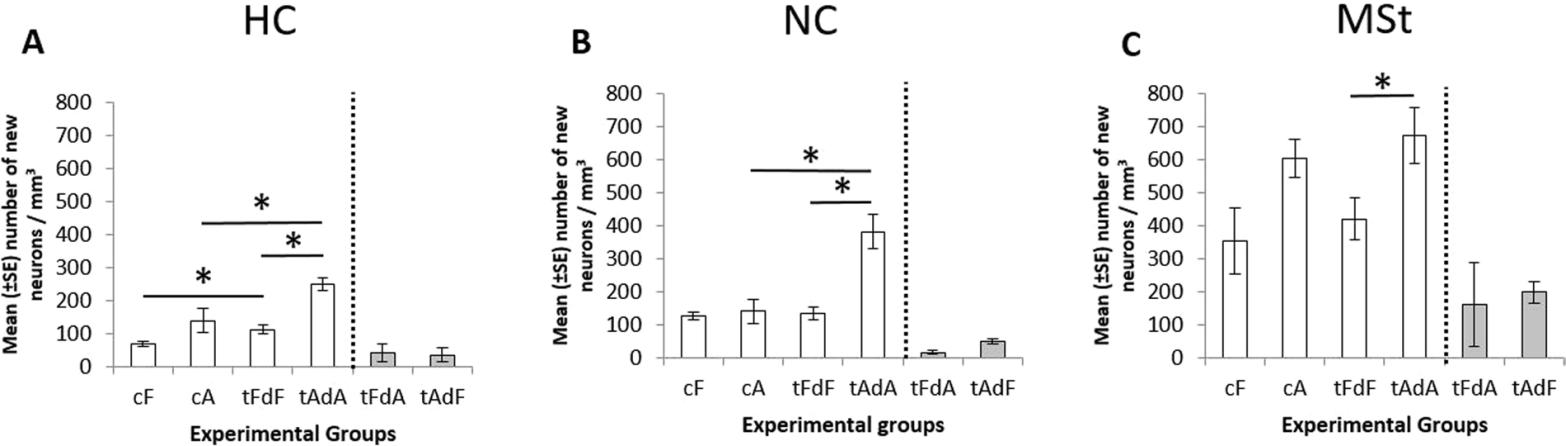
Mean number (±SE) of new neurons per mm^3^ in the six experimental groups in each of the three investigated brain regions. Abbreviations for experimental groups: cF - control February; cA - control August; tFdF - artificial conditions mimicking those in cF; tAdA - artificial conditions mimicking those in cA; tFdA – a “mixed” group, of February temperature and August day length; and tAdF – a “mixed” group, of August temperature and February day length. Abbreviations for brain regions: HC - hippocampal complex, NC - nidopallium caudale, MSt – medial striatum. *indicates a significant difference (p < 0.05). Dashed lines indicate the separation of the two “mixed” groups from the other groups (natural and artificial conditions).

Overall, levels of new neuronal recruitment (presented as mean numbers of BrdU labeled neurons per mm^3^ ± SE; Fig. 4), with values in the different regions (HC, NC, MSt) serving as repeated measures, differed significantly among the six experimental groups (F_(5,23)_ = 15.3, p < 0.001). Post-hoc comparisons indicated that neuronal recruitments in the two “mixed” groups (i.e., those with the combination between the two seasons) were lower than in most of the other four groups. More specifically, neuronal recruitment in tFdA was significantly lower than all groups except tAdF, whereas that in tAdF was significantly lower only from the August groups (cA and tAdA). The two mixed groups did not differ from each other. Because the two “mixed” groups (tFdA, tAdF) were different from most of the other groups, and because they represent unnatural combinations of temperature and day length, we excluded these two groups in the following assessment of seasonal effects:

Analysis of the overall effect of season (August/February) and location (indoor/outdoor) in a two-way repeated measures ANOVA showed that neuronal recruitment was higher during August (cA and tAdA) than during February conditions (cF and tFdF), with F_(1,16)_ = 25.34, p = 0.0001 (Fig. 4), and higher under the artificial indoor conditions (tFdF and tAdA) than under the natural outdoor conditions (cF, cA; F_(1,16)_ = 10.37, p = 0.0053; Fig. 4). Neuronal recruitment differed significantly among the brain regions (F_(2,19.9)_ = 60.23, p < 0.0001), with the highest recruitment in the MSt and the lowest in the HC.

No significant interactions were found between season and location, between season and brain region, or between location and brain region. However, the three-factor interaction of season X location X brain region was significant (F_(2,19.9)_ = 3.8, p = 0.04), indicating that the interaction between season and location varies across the brain regions. Therefore, two-way ANOVAs were conducted for each brain region separately. Again, neuronal recruitment was higher during August than during February conditions in all brain regions (F_(1,15)_ = 20.49, p = 0.0004 for HC; F_(1,16)_ = 12.91, p = 0.0024 for NC; and F_(1,16)_ = 10.18, p = 0.0057 for MSt), and higher under artificial indoor conditions than under natural outdoor conditions in the HC and NC (F_(1,15)_ = 12.71, p = 0.0028; F_(1,16)_ = 12.82, p = 0.0025, respectively), but not in the MSt. In addition, a significant season X location interaction was found in the NC (F_(1,16)_ = 11.31, p = 0.004).

Analysis of the effect of season for each location and brain region separately revealed that neuronal recruitment was significantly higher in all brain regions during August than during February under artificial indoor conditions (tAdA vs. tFdF) but not under natural outdoor conditions (cA vs. cF). However, the effect of location for each season and brain region separately was significant during August conditions (tAdA vs. cA) in the HC and NC brain regions but not in the MSt, and significant during February conditions (tFdF vs. cF) in the HC and not in the other two brain regions (indicated by * in Fig. 4).

These results indicate the following:

No seasonal effect on neuronal recruitment under natural outdoor conditions: A comparison between the groups that were kept under natural conditions revealed no seasonal effects on neuronal recruitment, in all the three tested brain regions (cF vs. cA).

A seasonal effect on neuronal recruitment under artificial indoor conditions: A comparison between the groups that were kept under artificial indoor conditions and imitated natural seasons (tAdA vs. tFdF) revealed a seasonal effect, with significantly higher levels of neuronal recruitment during August compared to February, in all the brain regions tested.

Natural outdoor conditions and similar artificial indoor ones have differential effects on neuronal recruitment: We found that levels of neuronal recruitment under artificial indoor conditions were significantly higher than those observed under natural and outdoor ones, during both August and February in the HC and during August in the NC. No such differences were observed during February in the NC as well as in the MSt during both seasons.

## Discussion

We aimed to study, for the first time in birds, in a controlled and systematic manner, whether there is a seasonal neuronal recruitment pattern that is not correlated to behavior, and if so – whether this pattern exhibits fewer neurons that are incorporated into brain regions in early spring than in late summer. To date, only little evidence supports this possibility: for example, Tramontin & Brenowitz^52^ found that wild song sparrows show higher neuronal recruitment in the HVC in the fall than in the spring, even though they retain the same song throughout adulthood. From other animals there is also relatively little information about this question. For example, in rodents there are indications that temperature (hot climate) is correlated with higher HC neuronal recruitment^53,54^, while in sheep a seasonal effect has been demonstrated on hypothalamic neurogenesis, which is modulated by photoperiod^55^. In turtles there is a temperature-dependent effect on cell proliferation, with higher new neuronal densities in warmer temperatures than in colder ones^56^. In lizards, a photoperiod effect on neurogenesis was recorded, with highest rates in spring compared to autumn^57,58^, while in snakes an opposite pattern was found^59^. However, all these examples can still be interpreted in the contexts of seasonal behaviors, particularly in relation to reproduction, and most of them did not separate day length from temperature.

### Lack of a seasonal effect on neuronal recruitment under natural outdoor conditions

Our data suggest that, under natural photoperiod and temperature conditions, without any association with behavioral patterns, there is no seasonal pattern in new neuronal recruitment, as no differences were found in neuronal recruitment in any of the three investigated brain regions when we compared two opposite seasons – August (cA) and February (cF). This implies that in the absence of behavioral, social or spatial changes, neuronal recruitment is not affected by the seasons of the year. We can assume that a similar output would occur also in Australia, in the natural habitat of zebra finches, because there they encompass diverse climatic zones, including tropical and subtropical types^17^, which are even milder than the Israeli climate, where our study had been conducted. Nevertheless, it can definitely be interesting to conduct a similar experiment in Australia and test this hypothesis.

We are aware of the fact that we need to be cautious when interpreting our results. For example, it could be that although we did not find any seasonal effect on densities of new neurons, a seasonal effect on overall neuronal recruitment could still exists, if the volumes of the investigated brain regions (which we did not measure in the current study) show seasonal variation. However, this is unlikely, because in a previous study, in which we kept zebra finches outdoors for 1, 2, and up to 5 months, we did not find any variation in the volumes of both the HC and NC^60^. In addition, in another study^61^, in which we compared neuronal recruitment between breeding and non-breeding zebra finches, we also did not find any changes in volumes of these brain regions. Another possible explanation for the lack of a seasonal effect in new neuronal recruitment that we have found in zebra finches could be related to the fact that they are non-seasonal birds (in relation to various behaviors, such as breeding, singing, diet, etc., as described in the Introduction). Hence, it is important to further test this question also in a seasonal songbird, for example the canary (*Serinus canaria domestica*), which can be easily studied. A comparison between seasonal and non-seasonal species will enable to conclude whether our finding, of a constant basic neuronal recruitment across seasons, is a general and broad phenomenon, or restricted only to non-seasonal birds. If our findings occur in other species, it will provide further support to the notion that had often been expressed in previous studies, including ours, as detailed in the Introduction, that seasonal neuronal recruitment is correlated with seasonal behavior or change, and enables organisms to adapt to their changing environment.

### A seasonal effect on neuronal recruitment under artificial indoor conditions

Surprisingly, when we tested the same question of seasonal neuronal recruitment under artificial conditions, we observed a different outcome than the one obtained under natural conditions. This time we found a seasonal pattern, with higher levels of neuronal recruitment in August (tAdA) compared with those recorded in February (tFdF). The difference was significant in all the three investigated brain regions.

The direction of this difference under artificial indoor conditions, i.e. of higher neuronal recruitment in August than in February, resembles that from other studies, which correlated it to behavior: neuronal recruitment into HC was highest in late summer/early fall in food-storing chickadees^8^, in migrating warblers^6^, and in HVC of singing canaries^62^. Moreover, a similar seasonal difference was also found in non-migrating warblers^6^, and in the song nucleus HVC of wild sparrows, despite the fact that in this species, unlike canaries, males retain the same song throughout adulthood^51^. Nevertheless, it is fair to note, as already mentioned in the Introduction, that in regards to food-storing behavior, others have found other results (reviewed by Sherry and MacDougall-Shackleton^11^).

It is not clear why outdoor and indoor conditions yielded different results. It could be suggested that the subtle and non-significant trend of higher recruitment in August than in February that occurs under outdoor conditions (cA vs. cF) becomes more pronounced and hence significant under indoor conditions (tAdA vs. tFdF) in all the three investigated brain regions. In any case, the overall evident difference between the results that were obtained under natural vs. artificial conditions could be due to inherent differences between the two settings. This is because, by definition, the differences between the two months that we tested (August and February) were sharper and more evident under artificial conditions, at least in two aspects. Firstly, the temperature in the indoor setup was kept constant, unlike the natural situation, in which there are always temperature fluctuations between days during the tested time period, as well as fluctuations within the 24-hour period, with a natural temperature drop during the night. Secondly, under natural conditions there is a gradual light-dark transition between day and night, both at sunrise and sunset. This transition, which might be behaviorally and physiologically important, was completely absent in our artificial conditions. Hence, the differences in neuronal recruitment patterns between indoor and outdoor conditions should be taken into consideration when planning indoor experiments, and they further emphasize the importance of conducting experiments under natural conditions. The need to be cautious in interpreting results that are obtained from indoor experiments is also demonstrated by the enhanced levels of neuronal recruitment that were recorded in both NC and HC under August indoors conditions and in the HC also during February conditions, so that they were significantly higher than those observed under the respective outdoor conditions. Taken together, these differences between indoor and outdoor indicate that we should be aware of the risk or possibility that data obtained from experiments that are conducted under indoor and artificial conditions might not accurately represent the natural situation.

### Artificial indoor conditions induce differential effects on neuronal recruitment in the investigated brain regions

Although neuronal recruitment in the HC and NC was affected by location (in the HC there was a significant difference between outdoor natural conditions and indoor artificial ones in both seasons and in NC only in August), neuronal recruitment in the MSt was not affected by location, in both seasons. Hence, MSt seems to be more resilient to the effect of artificial indoor conditions, maybe due to its highest rates of neuronal recruitment compared to HC and NC, which might moderate the effect of artificial conditions. Nevertheless, it is not clear why artificial August conditions had a more pronounced effect on neuronal recruitment (both in HC and NC), compared to those of artificial February (only in HC).

The relative resilience of neuronal recruitment in MSt under artificial indoor conditions is intriguing. MSt was formerly known as Lobus Paraolfactorius (LPO), but because this region contains mainly striatal cellular characteristics, it nomenclature had been revised into Medial Striatum^35^. Nevertheless, it is important to note that MSt lacks the one-to-one homology to the medial part of the mammalian striatum^63^. As suggested by lesioning experiments to the mammalian medial striatum, this area may have a crucial role in behavioral flexibility^64–66^. In birds, there is evidence for its role in associative learning, involvement in the rewarding effects of morphine and processing of object feature information^37,38,67,68^. Another study has indicated that inactivation of dorsomedial striatum of rats impaired behavioral flexibility that involved shifts between response and visual cues discrimination^69^. A similar link to visual perception was found in pigeons, when MSt lesioning caused cognitive rigidity that was expressed by impaired ability to discriminate based on color^36^. Sicard *et al*.^70^ suggested that MSt is photosensitive and is involved in the regulation of gonadotrophin secretion, however that study was conducted on quails that are known to be seasonal breeders. Thus, a possible explanation for the relative resilience pattern in neuronal recruitment in MSt may be due to a lack of seasonal significance in this area when there is no correlation to behavior. It could be that in non seasonal birds such as our zebra finches, which breed throughout the year, high visual vigilance for mate recognition is required regardless of the season.

### “Mixed” seasonal conditions significantly reduce neuronal recruitment

An important outcome of our study is that “mixing” temperature and day length (i.e. combinations between the two seasons; groups tAdF and tFdA) yielded significantly lower neuronal recruitment rates in the three examined brain regions. Originally, the purpose of these two groups was to test whether one of these seasonal cues is more crucial for neuronal recruitment than the other. However, the similar low neuronal recruitment in both mixed groups suggests that a natural combination of both temperature and day length is important for neuronal recruitment. When these two factors are mixed, recruitment is significantly reduced, and in the case of tFdA it is even lower than the low neuronal recruitment that was observed under both outdoor and artificial February conditions (cF and tFdF, respectively). This outcome is along the same line as suggested by Stevenson *et al*.^71^, who present evidence that even slight seasonal disruptions affect the health and welfare of organisms. It is possible (although not proven) that by mixing the two major seasonal components - temperature and day length - we interrupted the internal clock in our birds, which in turn affected neuronal recruitment. It is known that stress decreases neurogenesis (reviewed in Schoenfeld and Gould^72^) and that disruption of circadian rhythm induces stress (reviewed in Koch *et al*.^73^). Therefore, it is plausible that the birds in the “mixed” groups underwent chronic stress due to the alteration of the seasonal components, and that, in turn, reduced neuronal recruitment in their brains.

While evidence indicates a correlation between circadian disruption and mood disorders (reviewed in Bedrosian and Nelson^74^), and also demonstrates the role of impaired neurogenesis in affective disorders (reviewed in Samuels and Hen^75^), we still miss evidence that link aberrations in these processes. In the light of a new study^76^ that provides the first indication of seasonal effects on human cognitive function, our “mixed” groups might propose such a possible link between circadian and/or seasonal disruption, neurogenesis and mood disorders. Even though the synchrony that we altered was between temperature and day length (seasonal disruption) and not time and day length as the cited above (circadian disruption), our data reinforce the significance of such synchrony between light and temperature, and might suggest potential translational implications. This question, of course, is still to be tested.

### Overview and conclusions

Here we show that under natural photoperiod and temperature conditions, and in the absence of behavioral, social or spatial changes, neuronal recruitment remains constant across seasons (cA vs. cF). This lack of seasonality in new neuron recruitment could be due to the a-priori non-seasonal nature of our model – the zebra finch. Hence, to conclude whether this is a general and broad phenomenon, it has to be tested also in seasonal species. Surprisingly, under artificial conditions, the same set-up yielded a different outcome, of higher neuronal recruitment in August compared to February (tAdA vs. tFdF). The different results between natural and artificial conditions could be because of two reasons: Firstly, indoor temperature was constant, unlike the natural situation where fluctuations occur between days and within the 24-hour period. Secondly, the natural gradual light-dark transition was absent in our artificial conditions. We believe that our findings indicate that one has to be aware of the possibility that data obtained from indoor experiments under artificial conditions might not accurately represent the natural situation. Moreover, our study shows that artificial indoor conditions differentially affect neuronal recruitment in various brain regions, and we discuss possible explanations of this effect.

Lastly, our data indicate that “mixing” temperature and day length (tAdF and tFdA) results in a significant reduction of neuronal recruitment, demonstrating the importance of the natural combination of temperature and day length. Taken together, our results support the review by Calisi and Bentley^77^, which presents examples for experiments performed in a laboratory environment that are sometimes different than findings from similar experiments performed in the “real world”. While the authors suggest that endocrinological and behavioral experiments are particularly susceptible to influence from the environmental set-up, our study adds another dimension to this conclusion, by showing that neuroplasticity also changes under natural vs. artificial conditions. Another example for the importance of understanding how changes in housing conditions affect the question under investigation is the study by Dickens & Bentley^78^ who showed remarkable differences in breeding behavior and hypothalamic pituitary-adrenal activity between European starlings (*Sturnus vulgaris*) that were housed outdoors or indoors. We join in with the conclusion of these authors that experiments in both lab and field are needed when looking at complex biological systems.
